# Molecular-level architectural design using benzothiadiazole-based polymers for photovoltaic applications

**DOI:** 10.3762/bjoc.13.87

**Published:** 2017-05-10

**Authors:** Vinila N Viswanathan, Arun D Rao, Upendra K Pandey, Arul Varman Kesavan, Praveen C Ramamurthy

**Affiliations:** 1 Department of Materials Engineering, Indian Institute of Science, Bangalore, Karnataka, India; 2 Interdisciplinary Centre for Energy Research, Indian Institute of Science, Bangalore, Karnataka, India

**Keywords:** bulk heterojunction, donor–acceptor–donor polymer, low band gap polymer, organic photovoltaics

## Abstract

A series of low band gap, planar conjugated polymers, **P1** (PFDTBT), **P2** (PFDTDFBT) and **P3** (PFDTTBT), based on fluorene and benzothiadiazole, was synthesized. The effect of fluorine substitution and fused aromatic spacers on the optoelectronic and photovoltaic performance was studied. The polymer, derived from dithienylated benzothiodiazole and fluorene, **P1**, exhibited a highest occupied molecular orbital (HOMO) energy level at −5.48 eV. Density functional theory (DFT) studies as well as experimental measurements suggested that upon substitution of the acceptor with fluorine, both the HOMO and lowest unoccupied molecular orbital (LUMO) energy levels of the resulting polymer, **P2**, were lowered, leading to a higher open circuit voltage and short circuit current with an overall improvement of more than 110% for the photovoltaic devices. Moreover, a decrease in the torsion angle between the units was also observed for the fluorinated polymer **P2** due to the enhanced electrostatic interaction between the fluorine substituents and sulfur atoms, leading to a high hole mobility. The use of a fused π-bridge in polymer **P3** for the enhancement of the planarity as compared to the **P1** backbone was also studied. This enhanced planarity led to the highest observed mobility among the reported three polymers as well as to an improvement in the device efficiency by more than 40% for **P3**.

## Introduction

The great interest in organic photovoltaic (OPV) devices is motivated by their ease of low-temperature solution processing, light weight, flexibility and potential to produce large area devices [1]. The introduction of an interpenetrating donor and acceptor architecture in the active layer of the OPV devices led to a new type of device, the so-called bulk heterojunction (BHJ) solar cells, with improved power-conversion efficiency (PCE) [2–4]. A large number of polymer semiconducting materials of donor–acceptor–donor (D–A–D) architecture have been synthesized and used in OPV devices recently reaching remarkable PCEs of up to 11.7% [5–7]. However, the diversity of monomeric units and the numerous available reports on the structural complexity of D–A–D-conjugated p-type polymers indicate that there is still need for new materials which can further improve the performance of OPV devices based on D–A–D polymers [8–13]. The properties of D–A–D-type materials such as band gap, structural planarity, charge carrier transport, etc., can be easily tailored by careful selection, combination, and position of the donor and acceptor moieties.

For OPV systems, it is desirable that p-type polymers should have a low band gap for a broad absorption area of the solar spectrum to harvest a maximum number of photons [14]. Simultaneously, these compounds should also be soluble in common organic solvents and have good film forming properties. However, these are not the only parameters to consider for the design of a new donor polymer system. In OPV devices, a bicontinuous layer of a donor and an acceptor material is sandwiched between two electrodes. After the absorption of light, excitons are generated which dissociate towards the interface of the donor–acceptor layer and are separated into free carriers. These free charge carriers are then collected at the electrode for current generation [15]. The driving force for this charge separation originates from the energy offset between the frontier molecular energy levels of the donor and acceptor material, broadly known as the binding energy [15]. While reducing the band gap by adjusting the highest occupied molecular orbital (HOMO) and lowest unoccupied molecular orbital (LUMO) energy levels, a downhill driving force for exciton dissociation should be maintained for optimum performance of the OPV. If not, the total exciton dissociation will decrease, and hence, the overall device efficiency too.

Moreover, for efficient OPV systems, a moderately high charge carrier mobility is required, which is attainable by increasing the crystallinity of polymers with firmly packed polymer chains. However, an increase in polymer crystallinity will simultaneously decrease their processability in solution. This will result in the reduced formation of the desired bicontinuous morphology with the acceptor [16]. Hence, when designing new molecules for OPV applications, a subtle balance between lowering the band gap, crystallinity, and solubility should be maintained. Extensive studies have been reported for the tuning of optoelectronic and photovoltaic properties by architectural design at the molecular level [17–19], such as quinoidation of the polymer backbone [20], alternate D–A–D architectures [21–22], and substitution with electron-withdrawing or electron-donating groups [23–24]. Substitutions can be used to tune the band gap, energy levels, solubility, packing of material and morphology [8]. Among them, the introduction of fluorine has attained great interest because of its small size and strong electron-withdrawing nature, and fluorine substitution will amend both the HOMO and LUMO energy levels. In addition, substitution along the backbone persuades more inter- and intramolecular interactions [25–29]. Furthermore, the modification of π-bridges between the donor and acceptor unit of p-type molecules plays a significant role in increasing the efficiency for OPVs [30–31]. However, fused π-bridges (such as thienothiophene) having a larger molecular structure and higher degree of conjugation are less explored with respect to thiophene and furan spacers. Thienothiophene ensures a highly delocalized electron system and higher charge carrier mobility due to its rigid and coplanar fused structure. Also, some thienothiophene-based polymers show a noticeable bathochromic shift in their absorption spectra in comparison with thiophene-substituted polymers [32–34]. Herein, keeping all these criteria in mind, we endeavored to obtain a series of low band gap polymers, **P1**, **P2**, and **P3**, with matching HOMO–LUMO energy levels with the acceptor moiety, without sacrificing the planarity of the molecule. Benzothiadiazole and fluorene, which are commonly used moieties in D–A–D-type polymers, have been chosen as the acceptor and donor, respectively [35–36]. The acceptor motif was further coupled with thiophene to increase the conjugation length and absorption. The same acceptor moiety was substituted with fluorine and the effect of this substitution on the polymeric and photovoltaic properties was studied. Furthermore, the effect of planarity and conjugation extension on the polymer backbone was also studied by coupling with a fused thienothiophene moiety.

## Results and Discussion

**Scheme 1:**
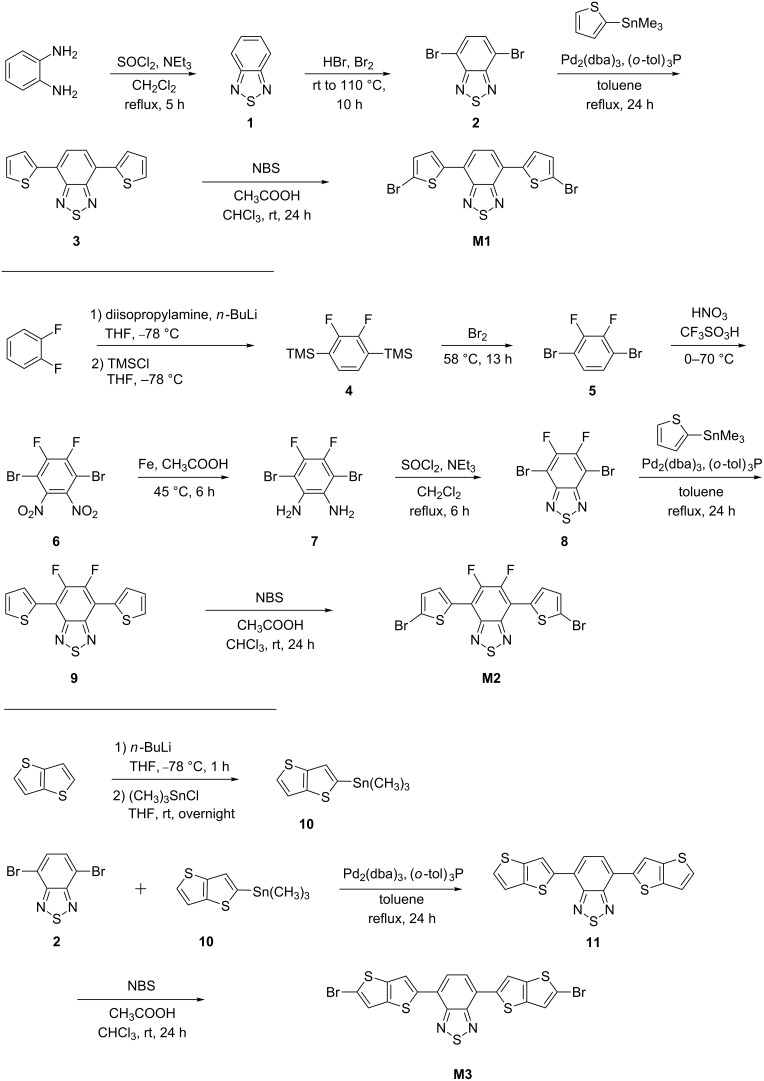
Synthetic route towards monomers **M1**, **M2** and **M3**.

For the synthesis of polymers with alternating donor–acceptor–donor architecture, suitable monomers were first prepared (Scheme 1). The synthesis of monomer **M1** started with the cyclization of *o*-phenylenediamine with thionyl chloride in the presence of triethylamine, a strong base and dichloromethane as the solvent at 0 °C. Compound **1** was then treated with bromine and HBr to obtain 4,7-dibromobenzo[*c*][1,2,5]thiadiazole (**2**). The latter compound was then coupled with trimethyl(thiophene-2-yl)stannane through a Stille reaction using tris(dibenzylideneacetone)dipalladium(0) and tri-*o*-tolylphosphine as the catalyst system. The dithiophenylated product **3** was washed several times with methanol to remove the palladium catalyst and other impurities and subsequently brominated using *N*-bromosuccinimide to produce the desired monomer **M1**.

The synthesis of fluorinated monomer **M2** started from 1,2-difluorobenzene. However, the direct bromination of this compound in the 1,4-position is hindered due to the electronegative fluorine substituents. Hence, 1,2-difluorobenzene was reacted with trimethylsilyl chloride in the presence of lithium diisopropylamide to afford the 1,4-disilylated intermediate **4** and bromination of the latter compound in neat bromine afforded the desired 1,4-dibromo-2,3-difluorobenzene (**5**). Nitration of **5** by treatment with fuming nitric acid and acetic acid gave dinitro compound **6**. The nitro groups in **6** were then reduced by treatment with iron powder and acetic acid. The cyclization of the diamino compound **7** (as described for compound **1**) afforded the difluorinated benzothiadiazole **8**. The monomer **M2** was obtained by coupling **8** with stannylated thiophene, followed by bromination using NBS. For the synthesis of monomer **M3**, the required thienothiophene substrate **10** was prepared by the butyllithium-mediated reaction of thieno[3,2-*b*]thiophene with chlorotrimethylstannane. The latter compound was then coupled with benzothiadiazole **2** affording compound **11**. Finally, bromination of **11** using NBS afforded the desired monomer **M3**.

**Scheme 2:**
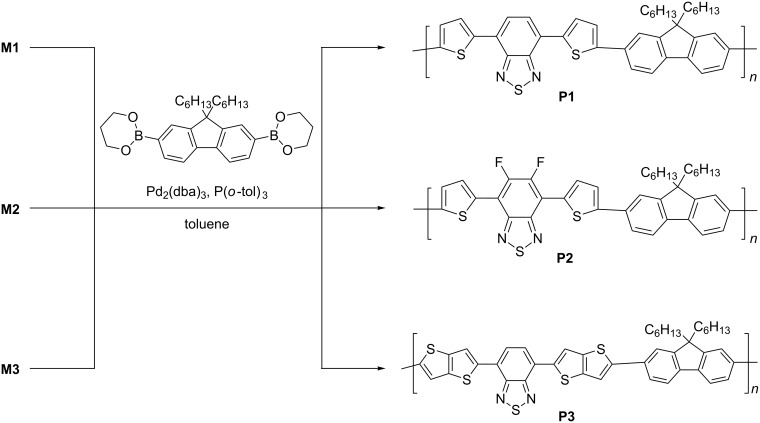
Synthesis of polymers **P1**, **P2**, and **P3**.

With the monomers **M1**–**M3** at hand, the corresponding polymers of D–A–D architecture were then synthesized. Thus, the brominated acceptor moieties **M1**–**M3** were reacted with 9,9-dihexylfluorene-2,7-diboronic acid bis(1,3-propanediol)ester under Suzuki coupling reaction conditions in the presence of sodium bicarbonate solution with tris(dibenzylideneacetone)dipalladium(0) and tri-*o*-tolylphosphine as the catalyst (Scheme 2). For experimental details, see Supporting Information File 1. The crude polymers were precipitated with methanol and subsequently treated with *N*,*N*-diethyl phenylazothioformamide in chloroform to remove any palladium impurities. Furthermore, unreacted reagents and oligomers were removed by successive Soxhlet extraction with methanol, hexane, and chloroform, respectively. The chloroform-soluble fraction of the polymers was concentrated and precipitated from methanol. The number-averaged molecular weight (*M*_n_), weight-averaged molecular weight (*M*_w_) and polydispersity index (PDI) of the polymers were calculated and are summarized in Table S1 (see Supporting Information File 1). All polymers showed excellent thermal stability with onset decomposition temperature of >410 °C.

**Figure 1:**
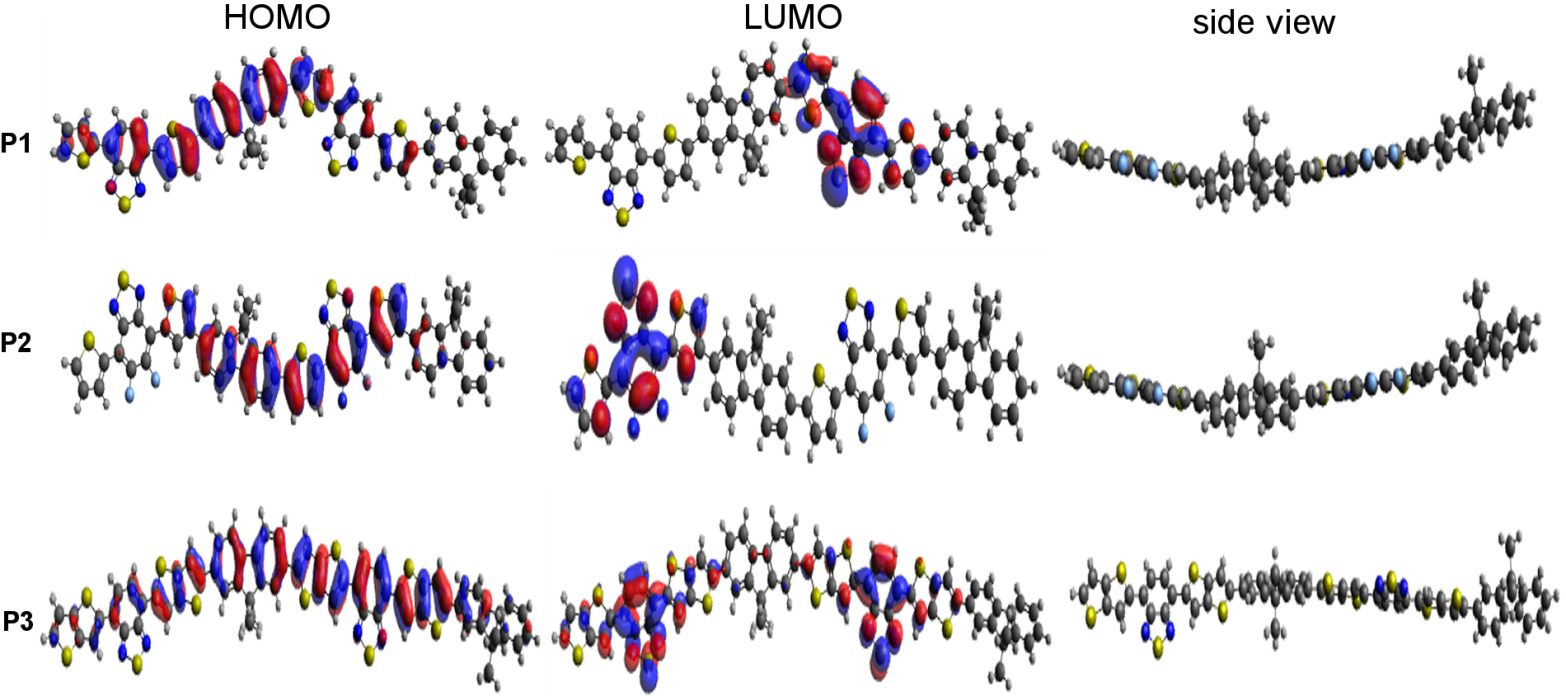
Isodensity surface plots of frontier molecular orbitals and optimized molecular geometries of **P1**, **P2** and **P3** and their HOMO–LUMO orbitals obtained from DFT calculations.

Computational modeling of the three polymers was performed to gain insight into the molecular energy levels along with band gaps using density functional theory (DFT) at the B3LYP/6-31G (d,p) level as implemented in Gaussian-09 software [37]. For computational modeling of the polymers, the alkyl chains were replaced by methyl groups and only two repeating units of monomers were used to keep the calculations simple. The ground-state potential energy of all optimized structures in their stable local minima was obtained to find the HOMO and LUMO. The isodensity surface plots of the frontier molecular orbitals and optimized geometries of the polymers are shown in Figure 1.

The HOMO orbitals show a good delocalization of charge along the polymer backbone, while the LUMO orbitals are localized at the acceptor moieties. In the case of the fluorinated analogue **P2**, the LUMO energy decreased by 0.10 eV compared to **P1** (indicating an increased electron affinity upon fluorination) and the corresponding HOMO level stabilized by 0.24 eV. Hence, both molecular energy levels could be tailored by the introduction of fluorine. In the case of **P3**, the HOMO level remained unchanged and the energy of the LUMO slightly decreased as compared to **P1**.

Furthermore, to check the planarity of the polymers, we calculated the torsion angles of each unit of the polymers from the optimized structures and they were found to be close to 180°. The calculated torsion angles of the polymers are collected in Table 1 and the angles are pictured in Figure S1 of Supporting Information File 1.

**Table 1:** Dihedral angles between all units of the polymer backbones in polymers **P1**, **P2**, and **P3**.

Polymer	θ1 °	θ2 °	θ3 °	θ4 °	θ5 °	θ6 °	θ7 °

**P1**	156	173.8	175.4	155	155.9	174.6	168.4
**P2**	149	175	179.8	154.1	149.3	175.5	178.3
**P3**	154	179.2	179.8	154.7	154	177.4	179.5

**Figure 2:**
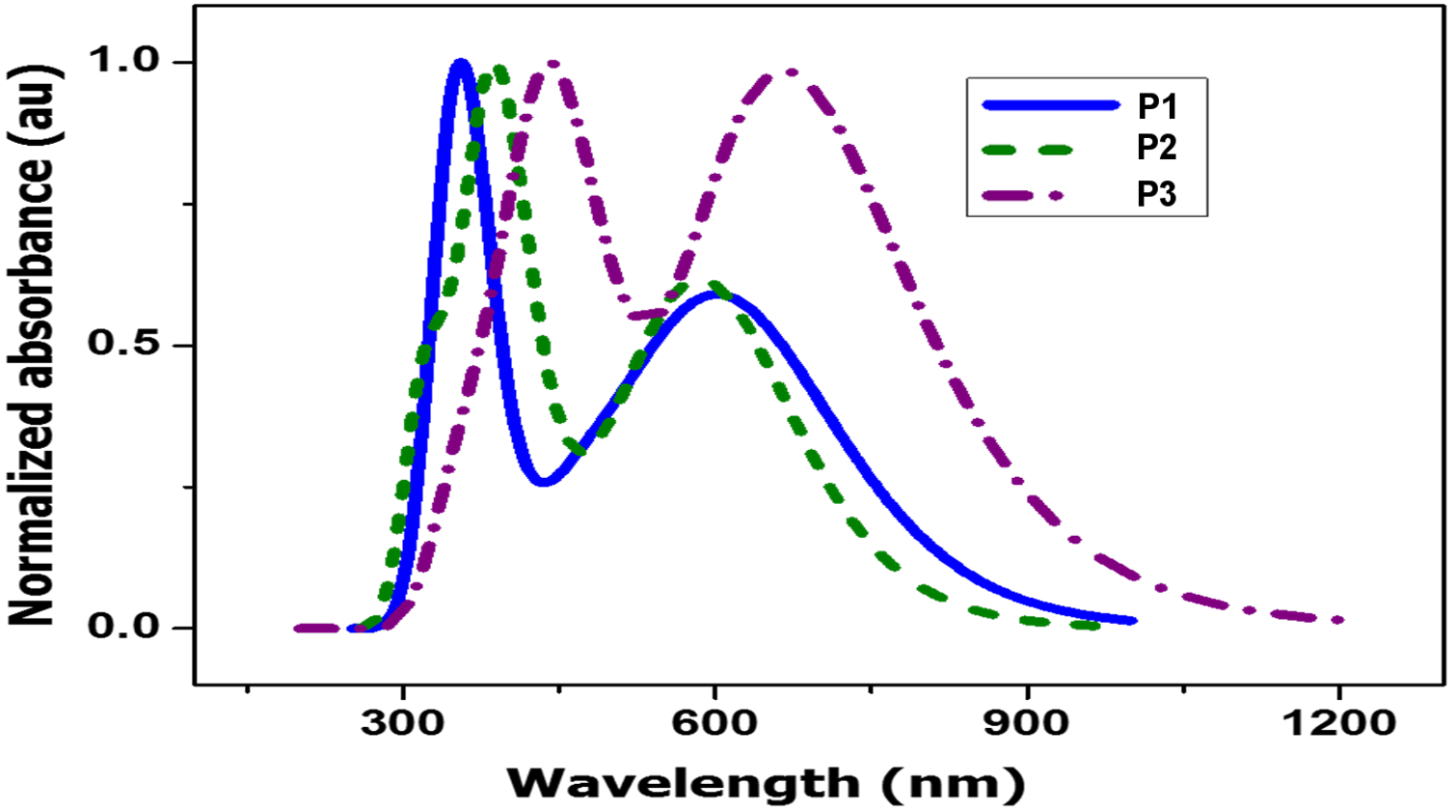
Theoretical absorption spectra of the polymers **P1**–**P3** calculated using TDDFT.

It is observed from the torsion angles for **P2** that the introduction of fluorine on the polymer backbone does not hinder the planarity. In contrast, it decreased the torsion angle between the fluorinated benzothiadiazole and the thiophene unit. This observation is attributed to the attractive electrostatic interaction between the positively charged sulfur atom and the negatively charged fluorine atom. In the case of **P3**, where the thiophene substituents are replaced by a fused thienothiophene bridge, a torsion angle of 179° is found between the benzothiadiazole and thienothiophene part. This clearly indicates an increased planarity of the polymer **P3** compared to **P1** due to the presence of the fused π-bridge. Moreover, time-dependent density functional theory (TDDFT) calculations were also performed to allow an estimation of the wavelengths at which electronic transitions take place upon excitation [37–38]. TDDFT calculations have been carried out using the B3LYP/6-31G (d,p) functional basis set to identify the first 30 electronic transitions in the polymers. The calculation shows that the first and most feasible singlet-to-singlet transition occurs at a wavelength of 656 nm, 601 nm, and 674 nm for **P1**, **P2**, and **P3**, respectively. In polymer **P1** this signal corresponds to the two electronic transitions from the HOMO to LUMO and HOMO−1 to LUMO energy levels. On the other hand, the transitions of polymer **P2** are attributed to HOMO to LUMO and HOMO to LUMO+1. The λ_max_ value of 674 nm calculated in **P3** includes three electronic transitions: HOMO to LUMO, HOMO−1 to LUMO, and HOMO to LUMO+1. The calculated absorption spectra for the polymers **P1**–**3** are shown in Figure 2 and are reliable with the experimental values obtained by UV–vis spectroscopy. Here it is observed that both the HOMO–LUMO values and the electronic transition wavelengths of the polymers showed promising results for OPV applications.

**Figure 3:**
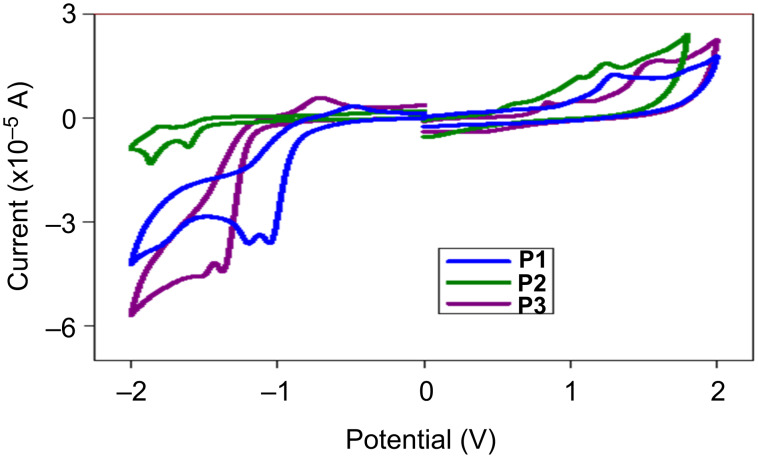
Electrochemical spectra of polymers **P1**–**P3**.

The HOMO–LUMO energy levels of the polymers were determined by cyclic voltammetry, using non-aqueous Ag/AgCl as the reference electrode in acetonitrile with 0.1 M tetrabutylammonium hexafluorophosphate as electrolyte at a scan rate of 100 mV/s. The instrument was calibrated with ferrocene/ferrocenium and was found to be ≈0.11 V. The HOMO–LUMO energy levels were calculated using the following equation based on the onset of oxidation and reduction obtained from the electrochemical spectra (Figure 3).

*E*_HOMO_ = −[*E*_ox (onset)_ − *E*_Fc/Fc_^2+^ + 4.8] eV

*E*_LUMO_ = −[*E*_red (onset)_ − *E*_Fc/Fc_^2+^ + 4.8] eV [39].

The effect of fluorine substitution at the polymer backbone is apparent on the frontier molecular orbitals of polymer **P2**. Both the HOMO and LUMO levels of the polymer have decreased energy as compared to polymer **P1** (from −5.48 eV to −5.53 eV and from −3.58 eV to −3.75 eV, respectively). This reduction of the HOMO energy level improves the resistance towards oxidative degradation of the material. Also, the reduction of the HOMO levels will further increase the open circuit voltage (*V*_oc_), since it is calculated from the difference of HOMO–LUMO energy levels of the donor and acceptor. In case of polymer **P3** the HOMO energy increased by 0.13 eV and the LUMO level remained almost the same for **P1**. This higher HOMO energy is due to the incorporation of the electron-donating thienothiophene bridge in the polymer backbone. The experimentally obtained values of frontier molecular energy levels follow the same trends with respect to the theoretical calculations. All polymers have deep-lying HOMO energy levels that are lower than the threshold for air oxidation (approximately −5.2 eV) [40–41] leading to a good ambient stability. The electrochemical band gap of the polymers, **P1**, **P2** and **P3** were determined as 1.9 eV, 1.78 eV, and 1.83 eV, respectively.

**Figure 4:**
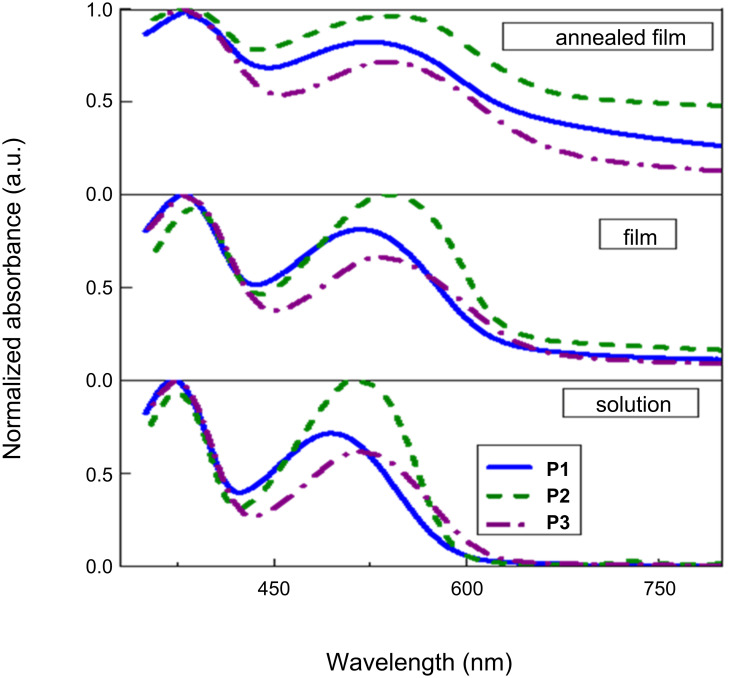
Normalized absorption spectra of the polymers in solution, film and annealed film (130 °C) forms.

Next, the optical properties of the polymers were studied by UV–vis spectroscopy. The absorption spectra were obtained in chlorobenzene solution (Figure 4). All polymers showed a broad absorption in the lower energy region (450–650 nm) due to the intramolecular charge transfer (ICT) through the backbone of polymers and another broad peak in the higher energy region due to π–π* transitions. A bathochromic shift in the λ_max_ of all thin film spectra of the polymers was observed, as compared to the solution spectra. This is due to the enhanced interchain stacking and ordered structural organization of the polymers in the thin film. The intensity of the ICT band of **P1** and **P3** is less than that of the π–π* transition band, whereas in **P2**, the ICT band is more pronounced due to the increased charge separation in the D–A–D polymer backbone due to the electronegative fluorine substituents in the acceptor moiety. As anticipated, the spectrum of **P3** is wider than the spectra of the other polymers since it has an extended conjugation along the backbone. The peak corresponding to the ICT of **P2** displays a red shift (≈33 nm) compared to the other polymers. This is caused by the increased electrostatic interaction between fluorine and sulfur in the solid state. The UV–vis spectra of annealed films of the polymers at 130 °C show an apparent red shift in the onset absorption because of an increased aggregation of the polymer chains upon heating. The optical band gaps of **P1**, **P2**, and **P3** were calculated from the onset of the absorption as 1.95 eV, 1.93 eV and 1.87 eV, respectively. Again, a lower band gap is observed for **P3** owing to the extended conjugation over the other polymers. The combined optical, electrochemical, and theoretical calculations are summarized in Table 2.

**Table 2:** Electrochemical and optical properties along with theoretical calculations.

Polymer	λ_max_ (solution) (nm)	λ_onset_ (solution) (nm)	λ_max_ (film) (nm)	λ_onset_ (film) (nm)	*E*_g_^a^ (film) (eV)	*E*_HOMO_ (eV)	*E*_LUMO_ (eV)	*E*_g_^b^ (eV)

**P1**	372, 498	598	377, 520	633	1.95	−5.48 (−4.9)^c^	−3.58 (−2.6)^c^	1.9 (2.2)^c^
**P2**	376, 510	596	386, 543	642	1.93	−5.53 (−5.1)^c^	−3.75 (−2.7)^c^	1.78 (2.3)^c^
**P3**	372, 515	617	382, 533	662	1.87	−5.35 (−4.9)^c^	−3.52 (−2.7)^c^	1.83 (2.1)^c^

^a^Optical band gap calculated from the absorption onset. ^b^Electrochemical band gap calculated from the cyclic voltammogram. ^c^Energy levels and band gap obtained from DFT calculations.

**Figure 5:**
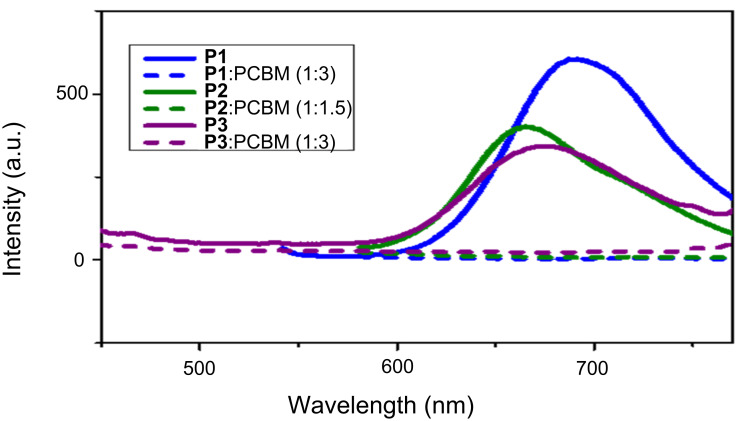
Photoluminescence spectra of polymers **P1**–**P3** and polymer:PC_70_BM blends.

Photoluminescence (PL) quenching studies were performed with pure donor polymers and with different ratios (by weight) of polymers with PC_70_BM ([6,6]-phenyl C_71_ butyric acid methyl ester) as an acceptor to evaluate the suitability of the polymers for photovoltaic devices. A significant reduction in the PL emission intensity of the donor when mixed with an acceptor is a good indication of an efficient charge transfer between the donor and acceptor when excited at the wavelength of the absorption maximum of the donor. An efficient charge transfer between donor and acceptor is essential for good solar cell devices. The PL spectra of the polymers, consisting of PC_70_BM with various blend ratios, are shown in Figure 5. Polymers **P1**, **P2**, and **P3** were excited at their absorption maxima of ≈377 nm, 543 nm and ≈395 nm, respectively. It is evident from the spectra that all polymers show significant quenching in their emission, indicating their suitability for OPVs.

**Figure 6:**
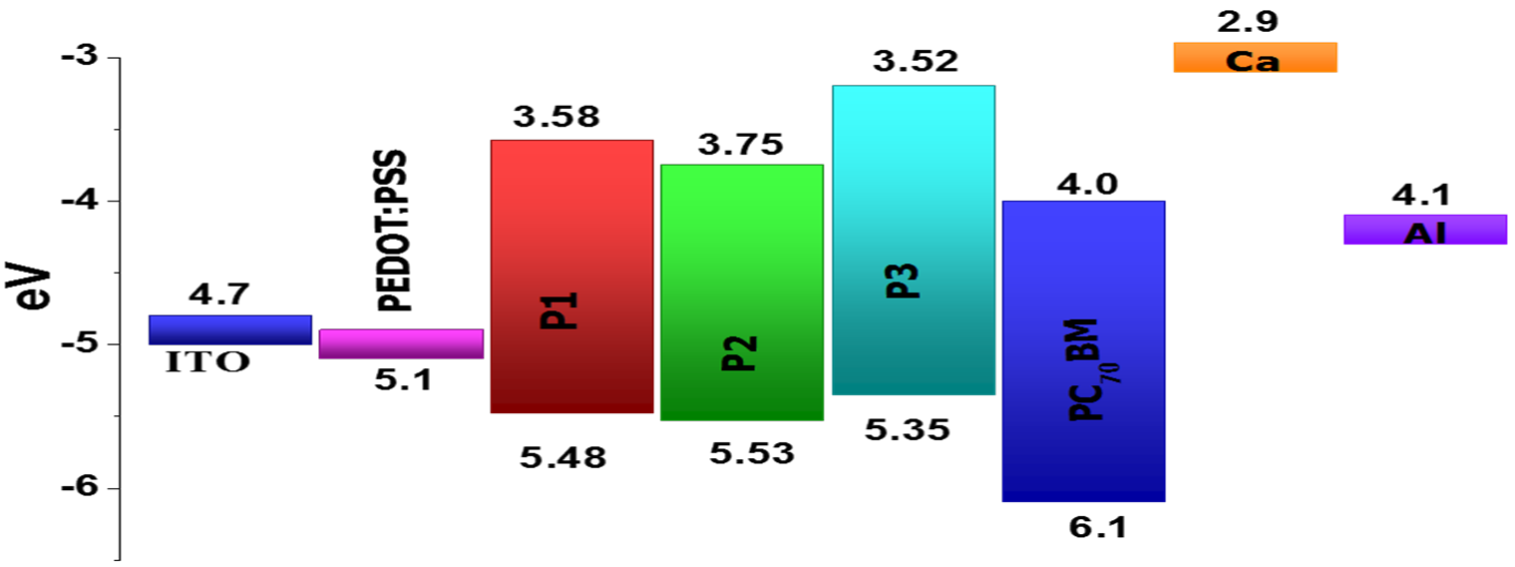
Bulk heterojunction solar cells device architecture, illustrating favorable conditions for absorption of sunlight.

Next, we fabricated OPVs based on bulk heterojunction (BHJ) solar cells of polymers **P1**, **P2** and **P3** and tested them with PC_70_BM as an acceptor. The device architecture of the BHJ solar cell was ITO/PEDOT:PSS (40 nm)/polymer:PC_70_BM (120 nm)/Ca (15 nm)/Al (100 nm). Figure 6 shows the favorable energy alignments for both electrons and holes for the collection at the electrodes once generated after absorption of sunlight.

**Figure 7:**
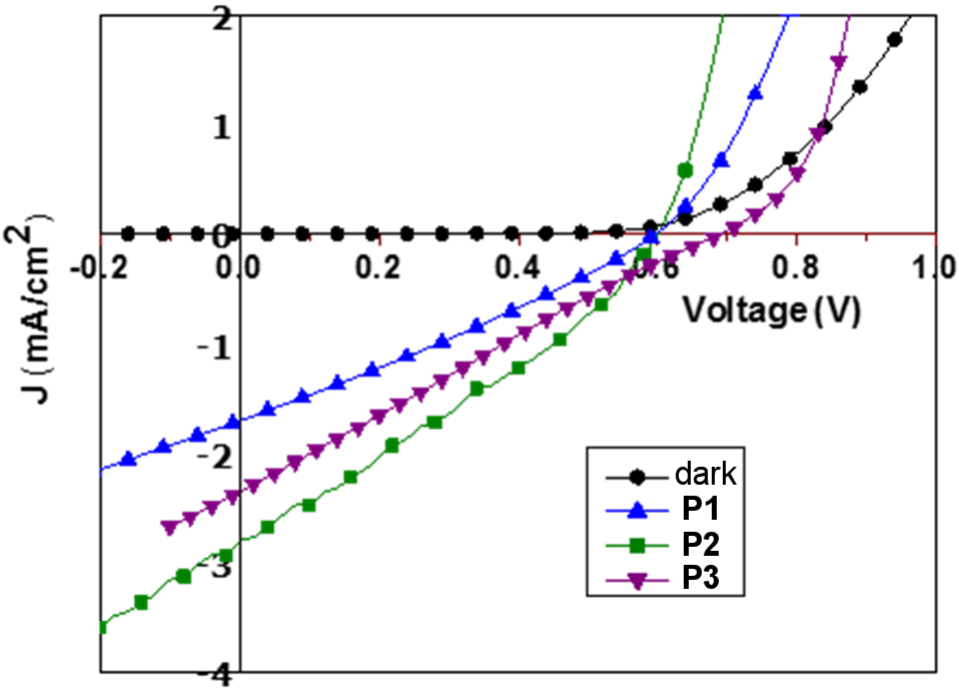
*J*–*V* Spectra in chlorobenzene (CB) for which the ratio of polymer:PC_70_BM was optimized as follows: **P1**:PC_70_BM, 1:3; **P2**:PC_70_BM, 1:1.5; **P3**:PC_70_BM, 1:3.

Figure 7 shows the current–voltage characteristics in the dark and after illumination with AM 1.5G (100 mW cm^−2^) for devices with **P1**, **P2,** and **P3**. The photovoltaic parameters of the devices are summarized in Table 3. Optimized blend ratios of 1:3, 1:1.5 and 1:3 were observed for polymers **P1**, **P2**, and **P3**, respectively.

**Table 3:** Photovoltaic properties of the devices based on polymers **P1**, **P2** and **P3**.

Polymer	Open circuit voltage (V)	Fill factor (%)	Short circuit current (mA/cm^2^)	η (%)	µ_h_ (cm^2^/V·s)

**P1**:PC_70_BM (1:3)	0.6	29	1.63 (1.41)^a^	0.28	1.32 × 10^−6^
**P2**:PC_70_BM (1:1.5)	0.62	35	2.8 (2.32)^a^	0.61	4.49 × 10^−5^
**P3**:PC_70_BM (1:3)	0.69	25	2.36 (2.17)^a^	0.41	3.98 × 10^−5^

^a^average *J*_sc_.

The blend **P1**:PC_70_BM 1:3 ratio shows a current density of 1.63 mA/cm^2^ and *V*_oc_ of 0.60 V. However, the device suffers from a moderate fill factor of 0.29. The corresponding device with the fluorinated polymer **P2** (having a deeper HOMO energy level) shows an improved *V*_oc_ (0.62 V) and *J*_SC_ (2.8 mA/cm^2^). Moreover, the device prepared with polymer **P3** shows a *J*_sc_ of 2.3 mA/cm^2^ and *V*_oc_ of 0.69 V (Figure 7). the hole mobilities (Table 3) of all the polymers were calculated using the space charge limited current method (SCLC, see Supporting Information File 1). Hole mobility values of 1.32 × 10^−6^ cm^2^/V·s, 4.49 × 10^−5^ cm^2^/V·s, and 3.98 × 10^−5^ cm^2^/V·s were observed for blends based on **P1**, **P2**, and **P3**, respectively. Compared to the device fabricated using **P1**, the corresponding devices fabricated with **P2** and **P3** show high hole mobilities. This increase in the mobility can be attributed to the enhanced planarity of the molecules. In the case of the fluorine-substituted polymer **P2**, an enhanced electrostatic interaction between the units was observed, and for **P3**, the fused thienothiophene moiety improves the planarity of the molecules.

To understand the photovoltaic results, we have also investigated the morphology of the active layers of the devices by tapping mode AFM. With this method, information about the topography as well as phase images as shown in Figure S2 (Supporting Information File 1) can be obtained. The active layer morphology in polymer solar cells is crucial and can drastically affect the performance of the devices. For optimum performance of an OPV device, the phase-separated donor and acceptor domain sizes should be twice the exciton diffusion length, which is typically of the order of 7–12 nm. From the images, a well-spaced, uniform phase contrast of donor polymer and acceptor was observed, indicating the uniform spatial distribution of the polymer:PC_70_BM in the matrix for all three polymers. Lower values of *J*_sc_ indicate that not all the photons absorbed are separated into free carriers, which could be attributed to the larger domain sizes of phase separated polymer and PC_70_BM. The roughness of **P1**, **P2**, and **P3** was obtained as 0.234 nm, 0.826 nm, and 0.914 nm, respectively.

**Figure 8:**
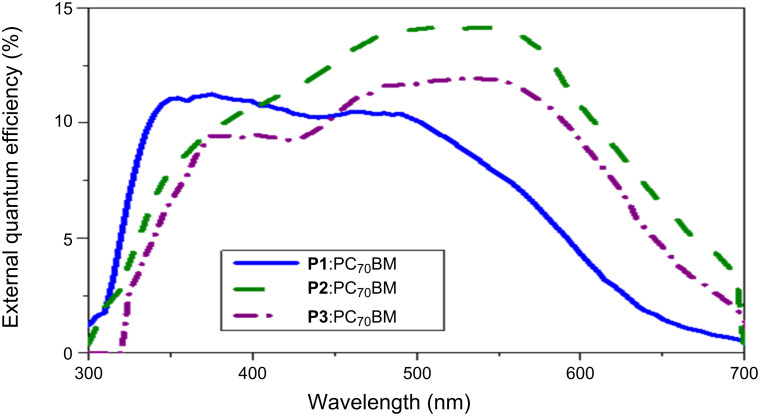
External quantum efficiency spectra of optimized devices fabricated with polymers **P1**, **P2** and **P3** and PC_70_BM as an acceptor.

The relatively low external quantum efficiency (EQE) values obtained for the reported polymers also explain the lower *J*_sc_ values. Basically, EQE spectra reveal the photon–current response of the devices, providing information about the number of charges contributing to the overall device current compared to the total number of incident photons at a particular wavelength. Figure 8 shows the EQE spectra for devices with **P1**, **P2**, and **P3**. The device based on **P2** shows an excellent photocurrent response over the absorption range of 320–700 nm, with a maximum at around 620 nm. Similarly, the devices fabricated with **P1** and **P3** show two distinct peaks at 350 and 470 nm (**P1**) and at 320 nm and 523 nm (**P2**), respectively. This implies that the overall photocurrent generation is contributed by the full polymer absorption range. By integrating the EQE spectra with the AM1.5G spectrum, the calculated *J*_sc_ values were obtained as 1.41 mA cm^−2^, 2.32 mA cm^−2^ and 2.17 mA cm^−2^ for blends with **P1**, **P2**, and **P3**, respectively (Table 3).

## Conclusion

In conclusion, we have synthesized three polymers with D–A–D architecture based on benzothiadiazole and fluorene. The effect of substitution with electron-withdrawing fluorine substituents and the incorporation of fused thienothiophene groups in the polymer backbone and their effect on the optoelectronics and photovoltaic performances has been demonstrated. It was observed that the incorporation of fluorine, a strong electron-withdrawing group, resulted in deeper HOMO energy levels for the polymer **P2** as compared to polymer **P1**. The fluorination also enhances the intramolecular interaction between the polymer chains, which is reflected in the higher hole mobility of **P2** over **P1**. Though the photovoltaic parameter values are very low for these polymers, it was observed that fluorination could increase the overall device performance by ≈110%. The effect of increased planarity along the polymer backbone was further explored by introducing the thienothiophene motif, a fused aromatic π-bridge in polymer **P3**, resulting in better stacking between aromatic units as compared to polymer **P1**. A bathochromic shift in the absorption spectra along with a higher hole mobility in the resulting polymer was observed. This resulted in an increase in the short circuit current from 1.63 mA/cm^2^ to 2.36 mA/cm^2^ with an increase in the overall efficiency by ≈46%. These studies suggest that planar-conjugated polymers based on flourene and benzothiadiazole (when substituted with appropriate groups) can play a vital role in attaining higher efficiency for D–A–D-based OPV systems.

## Experimental

The syntheses of the monomers and polymers are provided in the Supporting Information File 1. The fabrication of the photovoltaic devices has been carried out using the following procedure. Patterned ITO-coated glass substrates (Xinyan Technology Limited, Taiwan) were sequentially cleaned in deionized (DI) water with soap (Hellmanex III), DI water, isopropyl alcohol, acetone and water under sonication for ≈30 min each. The cleaned substrates were then dried with nitrogen gas. UV–ozone was performed on cleaned substrates for 30 min to remove residual impurities. PEDOT:PSS (Baytron VP Al 4083) was spin-coated on the UV–ozone-treated ITO substrate at 3000 rpm, for 60 s followed by annealing at 130 °C for 20 min to remove residual solvents. The PEDOT:PSS-coated substrates were then immediately transferred into the glove box (H_2_O <1 ppm, O_2_ <1 ppm) for active layer coating. The solutions with different blend ratios of polymers and PC_70_BM were prepared in chlorobenzene by weight ratio and stirred overnight at 60 °C in the dark. The concentrations of the solutions were kept at 25 mg/mL. The blend solution was then spin-coated on the PEDOT:PSS-coated substrate at 700 rpm for 60 s to obtain a film thickness of 120 nm. The substrates were then annealed at 150 °C for 15 min before being placed in the thermal evaporator for the cathode (Ca/Al) evaporation. Finally, 15 nm Ca (at a rate of 0.2 Å/s) and 100 nm Al (at the rate of 1 Å/s) were subsequently evaporated at a base pressure of 3 × 10^−6^ mbar to complete the devices. The thickness of the different layers was measured using a Dektak XT surface profiler.

## Supporting Information

File 1 Experimental details and characterization data.
