# Supplementary material for: Molecular-level architectural design using benzothiadiazole-based polymers for photovoltaic applications
Source: Beilstein J Org Chem. 2017 May 10;13:863–73. doi: 10.3762/bjoc.13.87 (PMC5433161; doi:10.3762/bjoc.13.87)
Supplement: File 1 — Experimental details and characterization data. [file Beilstein_J_Org_Chem-13-863-s001.pdf]

Supporting information

for

Molecular-level architectural design using benzothiadiazole-based polymers for photovoltaic applications

Vinila N. Viswanathan[1], Arun D. Rao[1], Upendra K. Pandey[2], Arul Varman Kesavan[1] and Praveen C. Ramamurthy[1,2*§]

Address: [1]Department of Materials Engineering, Indian Institute of Science, Bangalore, Karnataka, India and [2]Interdisciplinary Centre for Energy Research, Indian Institute of Science, Bangalore, Karnataka, India

Email: Praveen C. Ramamurthy - onegroupb203@gmail.com

[§]Fax: +91-80-2360-0472; Tel: +91-80-2293-2627.

* Corresponding author

Experimental details and characterization data

Contents:

Materials and methods

Synthesis of monomers

Table S1: MWD of polymers

Figure S1: Dihedral angles of all the polymers

Figure S2: Topographic images of polymer blends

Space charge limited current information

Figure S3: SCLC Hole mobility characteristics of polymers

Materials and methods

O-Phenylenediamine, tris(dibenzylideneacetone)dipalladium(0), tri-*o*-tolylphosphine, 2-(trimethylstannyl)thiophene, 9,9-dihexylfluorene-2,7-diboronic acid bis(1,3-propanediol)ester, 1,2-difluorobenzene, and trimethylsilylchloride, thieno[3,2-*b*]thiophene, were obtained from Sigma–Aldrich chemical company and used without further purification. Tetrahydrofuran (THF) was dried over sodium/benzophenone ketyl and distilled prior to use. *n*-BuLi (1.6 M in hexane) was obtained from Across Chemicals and used as received. Silica gel of 60–120, 100–200 mesh was used for column chromatography. All other reagents and solvents were obtained from commercial suppliers and used without further purification.

Proton nuclear magnetic resonance spectra were recorded using a Bruker NMR spectrometer with TMS as the internal standard as solutions in $CDCl_3$. Optical studies of the polymer were carried out from the absorption spectra measured using a Perkin-Elmer Lambda 35 spectrophotometer. Electrochemical properties were determined from cyclic voltammograms recorded using a CH660D CH instrument. 0.1 M Electrochemical grade tetrabutylammonium hexafluorophosphate was used with acetonitrile as the electrolyte. The electrochemical cell comprised three electrodes: Ag/AgCl as the reference electrode, platinum wire as the counter electrode and a glassy carbon electrode as the working electrode. The molecular weight of the polymers were determined by gel permeation chromatography (GPC) using a Waters GPC instrument with polystyrene as the standard and HPLC grade THF as the eluent. Thermal property studies were carried out by thermogravimetric analysis using a TGA 2950 thermal analyzer (TA instruments-STA 409 PC). Photoluminescence was measured using a Hitachi, Model No. F7000. Atomic force microscopy (AFM) was used to measure the surface roughness and phase contrast imaging using a Bruker ICON Scan Analyst. The current density–voltage (*J*–*V*) spectra were measured using a Keithley 4200 parameter analyzer and employed light intensity of 100 mW/cm^2 using Oriel Sol3A Class AAA simulator (intensities were calibrated using a standard Si solar cell). External quantum efficiency (EQE) was performed using an Enlitech setup (model QE-R) calibrated for 1 sun using NREL certified Si detector. The device fabrication has been carried out using Jacomex glove box integrated with Angstrom Engineering thermal evaporator, solar simulator and EQE set up.

Synthesis of monomers

2,1,3-benzothiadiazole (**1**)

In a similar manner as described in [1], a mixture of *o*-phenylenediamine (2 g, 18.52 mmol) in 20 mL DCM and triethylamine (10.3 mL, 74.1 mol) were stirred at room temperature until total dissolution of the diamine was observed. Thionyl chloride (2.69 mL, 37.3 mol) was added slowly to the reaction mixture via a syringe at 0 °C, the mixture stirred for 30 min at the same temperature followed by reflux for 5 h. Afterwards, the reaction mixture was concentrated under reduced pressure and brought to pH 1 using conc.HCl. The organic layer was extracted with DCM and then dried over anhydrous Na_2SO_4. The solvent was evaporated using a rotary evaporator under reduced pressure and the residue further purified by column chromatography using hexane as the eluent. Yield 90%, mp 44 °C. ^{1}H NMR spectrum (400 MHz, $CDCl_3$) δ (ppm) 7.99-8.03 (d, 2H), 7.23-7.61 (t, 2H)

4,7-Dibromo-2,1,3-benzothiadiazole (**2**)

As analogous to [1], 2,1,3-benzothiadiazole (**1**, 2 g, 14.69 mol) and 5 mL of HBr (48%) were placed in a 100 mL two-necked round-bottomed flask. A solution of Br_2 (1.5 mL, 29.4 mol) in HBr was added slowly through a dropping funnel at 0 °C. After completion of the addition, the mixture was stirred for 4 h at rt followed by reflux for 6 h. The formation of an orange solid precipitate was observed. The mixture cooled to rt and a sufficient amount of saturated solution of $NaHSO_3$ was added to remove excess bromine. The solid product was filtered off and thoroughly washed with DI water followed by diethyl ether. The product was purified by recrystallization from ethanol. Yield 83%, mp 188 °C. ^{1}H NMR spectrum (400 MHz, CDCl3) δ (ppm) 7.73 (d, 2H).

4,7-Dithienyl-2,1,3-benzothiadiazole (**3**)

As reported in [2], a solution of 4,7-dibromo-2,1,3-benzothiadiazole (**2**, 200 mg, 0.680 mmol) and 2-(tributylstannyl)thiophene (558.27 mg, 1.492 mmol) in 4 mL toluene was taken under nitrogen atmosphere. The reaction mixture was purged with N_2 for 15 min and tris(dibenzylideneacetone)palladium(0) (12 mg, 0.014 mmol) and tri(*o*-tolyl)phosphine (21.2 mg, 0.05 mmol) were added. After flushing nitrogen for 15 min the mixture was refluxed 110 °C for 24 h under inert atmosphere. The resultant mixture was cooled and precipitated from methanol. Yield 90%. ^{1}H NMR spectrum (400 MHz, CDCl3) δ (ppm) Ar H 8.132(dd, 2H, J = 8.5 Hz), 7.89(d, 2H), 7.46 (d, 2H, J = 5 Hz), 7.21 (d, 2H).

4,7-Bis(5-bromothiophen-2-yl)benzo(1,2,5)thiadiazole (**M1**)

Compound **3** (100 mg, 0.332 mmol) and 1.5 mL acetic acid and 3 mL of $CHCl_3$ were stirred at 0 °C under nitrogen purging for 10 min. NBS (130 mg, 0.732 mmol) was added in portions at the same temperature and the reaction mixture stirred for 24 h at rt under inert atmosphere. Then it was poured into water and extracted with dichloromethane. The organic phase was dried over anhydrous sodium sulfate and concentrated in a rotary evaporator under reduced pressure. Further purification of the monomer **M1** was carried out by column chromatography with hexane as the eluent. Yield: 60%. ^{1}H NMR spectrum (400 MHz, CDCl3) δ (ppm) Ar H 8.06 (d, 2H, J = 3.5 Hz), 7.8(s, 2H,), 7.1(d, 2H, J = 4 Hz).

2,3-Difluoro-1,4-bis(trimethylsilyl)benzene (**4**)

A solution of diisopropylamine (9.3 mL,65.78 mmol) in 100 mL dry THF was placed in a two-necked round-bottomed flask under nitrogen atmosphere and cooled to −78 °C. *n*-BuLi (41.1 mL, 65.78 mmol, 1.6 M solution in *n*-hexane) was added drop wise over 30 min and the reaction mixture stirred for 30 min at the same temperature. 1,2-Difluorobenzene (2.59 mL, 26.29 mmol) and trimethylsilylchloride (9.5 mL, 65.78 mmol) were added slowly through dropping funnel and the mixture stirred for 3 h at the same temperature. The reaction was quenched with water and extracted with ethyl acetate. The organic phase was dried over anhydrous sodium sulfate and concentrated using rotary evaporator to yield (95%) of the product as white solid. ^{1}H NMR spectrum (400 MHz, CDCl3) δ (ppm) Ar H 7.09(d, 2H), 0.32(18H).

1,4-Dibromo-2,3-difluorobenzene (**5**)

Bromine (9.9 mL) was placed in a 100 mL two-necked round-bottomed flask connected to a condenser and a gatt tube under ice cold condition. 2,3-Difluoro-1,4-bis(trimethylsilyl)benzene (**4**, 5 g, 19.34 mol) was added and the mixture stirred for 1 h 58 °C. A further portion of bromine (0.5 mL) was added to the mixture and stirring continued at the same temperature for 12 h. After cooling to 0 °C the reaction was quenched by the addition of sodium bicarbonate solution. The mixture was extracted with ethyl acetate, the organic phase washed with brine and dried over magnesium sulfate. The solution was then concentrated under vacuum using a rotary evaporator and the crude product purified by column chromatography. Yield (70%). ^{1}H NMR spectrum (400 MHz, CDCl3) δ (ppm) Ar H 7.23(m, 2H).

1,4-Dibromo-2,3-difluoro-5,6-dinitrobenzene (**6**)

To trifluoromethanesulfonic acid (43 mL) in a two-necked round-bottomed flask at 0 °C fuming nitric acid (2.13 mL) was added slowly and the mixture stirred for 30 min. 1,4-dibromo-2,3-difluorobenzene (**5**, 4.33 g, 15.91 mmol) was added in parts and the mixture stirred at rt for 2 h. The mixture was cooled to 0 °C and another 2.1 mL of fuming HNO_3 were added slowly and stirring continued overnight at 70 °C. After cooling to rt and the mixture was poured into ice cold water. After filtration and recrystallization from ethanol, the desired product was obtained as a white solid with a yield of 45%. ^{13}C NMR spectrum (100 MHz, CDCl3) δ (ppm) Ar C 151 (d), 148 (d), 141,105.

3,6-Dibromo-4,5-difluorobenzene-1,2-diamine (**7**)

1,4-dibromo-2,3-difluoro-5,6-dinitrobenzene (**6**, 800 mg, 2.21 mmol), iron powder (1.78 g) and acetic acid (32 ml) were placed in a two-necked round-bottomed flask and stirred at 45 °C for 6 h under a nitrogen atmosphere. The reaction mixture was allowed to cool and poured in 5% NaOH solution and extracted with ethyl acetate. The organic phase was washed with sodium bicarbonate solution, followed by brine and water. After drying over anhydrous sodium sulfate the solvent was removed under vacuum using a rotary evaporator to yield a fine orange-yellow solid with a yield of 72%.

4,7-Dibromo-5,6-difuorobenzo[*c*][1,2,5]thiadiazole (**8**)

3,6-Dibromo-4,5-difluorobenzene-1,2-diamine (**7**, 737 mg, 2.441 mmol) was added to a solution of 15 mL of dichloromethane and triethylamine (1.36 mL, 9.76 mmol) and cooled down to ice cold condition. Thionyl chloride (0.36 mL) was added very slowly using dropping funnel, the temperature of the reaction mixture was brought up to rt and subsequently refluxed at 46 °C for 6 h. The mixture was cooled to rt and was added to ice cold water. The pH was brought to ~1 using HCl. The organic part was extracted with dichloromethane and the organic layer washed with water and dried over sodium sulfate. The solvent was removed under vacuum and the crude product purified by column chromatography to obtain compound **8** as fine white needles. Yield 88%. ^{13}C NMR spectrum (100 MHz, CDCl3) δ (ppm) Ar C 153, 150.5 (d) 148.8 (t), 99.4 (d)

5,6-Difluoro-4,7-di(thiophen-2-yl)benzo[*c*][1,2,5]thiadiazole (**9**)

As reported in [2], a solution of 4,7-dibromo-5,6-difluorobenzo[*c*][1,2,5]thiadiazole (**8**, 340 mg, 1.03 mmol) and 2-(tributylstannyl)thiophene (0.657 mL, 2.06 mmol) in 4 mL

toluene was placed in a two-necked round-bottomed flask under nitrogen atmosphere. Nitrogen was purged through the reaction mixture for 15 min, followed by the addition of tetrakis(triphenylphosphine)palladium(0) (15 mg, 0.01mmol). After flushing with nitrogen for 15 min the mixture was refluxed for 24 h at 110 °C under inert gas atmosphere. The resultant reaction mixture was cooled and precipitated from methanol. Yield 90%. ^{1}H NMR spectrum (400 MHz, CDCl3) δ (ppm) Ar H 8.29(d, 2H, *J* = 3.6 Hz), 7.6(d, 2H, *J* = 5.5 Hz), 7.27(t, 2H, *J* = 4.2 Hz).

4,7-Bis(5-bromothiophen-2-yl)-5,6-difuorobenzo[*c*][1,2,5]thiadiazole (**M2**)

Compound **9** (290 mg, 0.862 mmol), 6.3 mL acetic acid and 10 mL of chloroform were stirred at 0 °C. The solution was purged with nitrogen for 10 min and NBS (460 mg, 2.58 mmol) was added portion wise to the mixture at same temperature. The reaction mixture was stirred at rt for 24 h under inert atmosphere and extracted with dichloromethane. The organic layer was dried over anhydrous sodium sulfate and concentrated using a rotary evaporator under reduced pressure. Further purification of the compound was done by column chromatography with hexane as the eluent. Yield: 60%. ^{1}H NMR spectrum (400 MHz, CDCl3) δ (ppm) Ar H 7.8(d, 2H, *J* = 4.2 Hz), 6.8(d, 2H, *J* = 3.6 Hz).

Trimethyl(thieno[3,2-*b*]thiophene-2-yl)stannane (**10**)

Thieno [3,2-*b*]thiophene (250 mg, 1.79 mmol) was dissolved in 3 mL dry THF under nitrogen atmosphere. Butyllithium (1.33 ml) was added dropwise at −78 °C and the solution further stirred for 1 h at the same temperature. Then trimethyltinchloride (2.13 mL) was added and the mixture kept stirring overnight at rt. Water was added to quenc the reaction and the mixture extracted with diethyl ether. The organic layer was dried over anhydrous magnesium sulfate and concentrated using a rotary evaporator to obtain the product as a colorless liquid with a yield of 80%.

4,7-Bis(thieno[3,2-*b*]thiophene-2-yl)benzo[*c*][1,2,5]thiadiazole (**11**)

A solution of 4,7-dibromo-2,1,3-benzothiadiazole (**2**, 388 mg, 1.31 mmol) and trimethyl-(thieno[3,2-*b*]thiophene-2-yl)stannane (**10**, 799.8 mg, 2.63 mmol) in 4 mL toluene was purged with nitrogen for 15 min. Then, tris(dibenzylideneacetone)palladium(0) (12 mg, 0.014 mmol) and tri(*o*-tolyl)phosphine (21.2 mg, 0.05 mmol) were added to the mixture. Nitrogen flushing was continued for 15 min and the mixture heated at 110 °C for 24 h under

inert atmosphere. The resulting mixture was cooled and precipitated from methanol. Yield 90%. ^{1}H NMR spectrum (400 MHz, CDCl3) δ (ppm) Ar H 8.5(S, 2H), 7.9(S, 2H), 7.47(d, 2H, *J* = 6 Hz), 7.321(d, 2H).

4,7-Bis(5-bromothieno[3,2-*b*]thiophene-2-yl)benzo[*c*][1,2,5]thiadiazole (**M3**)

The mixture of 4,7-bis(thieno[3,2-*b*]thiophene-2-yl)benzo[*c*][1,2,5]thiadiazole (**11**, 280 mg, 0.678 mmol) in 17.8 mL acetic acid and 6.69 mL chloroform was stirred at ice cold condition and purged with nitrogen for 10 min. Then, NBS (362 mg, 2.03 mmol) was added portion wise at the same temperature and the reaction mixture stirred at rt for 24 h under inert atmosphere. The reaction is quenched by the addition of water and the mixture extracted dichloromethane. The organic layer was dried over anhydrous sodium sulfate and concentrated using a rotary evaporator under reduced pressure. Further purification of the compound was carried out by column chromatography with hexane as the eluent. Yield: 60%. ^{1}H NMR spectrum (400 MHz, CDCl3) δ (ppm) Ar H 7.30 (d, 2H, *J* = 3Hz), 7.07 (d, 2H, *J* = 6 Hz), 6.59 (d, 2H).

General procedure for the synthesis of polymers by Suzuki coupling

P1

A mixture of 4,7-bis(5-bromothiophen-2-yl)benzo(1,2,5)thiadiazole (**M1**, 200 mg, 0.436 mmol) and 9,9-dihexylfluorene-2,7-diboronic acid bis(1,3-propanediol)ester (219.2 mg, 0.436 mmol) in 5 mL dry toluene was flushed with nitrogen for 15 min. Then, $Pd_2(dba)_3$ (13.7 mg, 0.015 mmol) and tri-(*o*-tolyl)phosphine (9.28 mg, 0.070 mmol) were added. The mixture was purged with nitrogen for 15 min and 2 M Na_2CO_3 solution was added. The solution was flushed with nitrogen again for 15 min and refluxed for 24 h under a nitrogen atmosphere. The reaction mixture was cooled and precipitated in methanol and the obtained polymer was dissolved in chloroform and *N*,*N*-diethyl-phenylazothioformamide (10 equiv to the palladium catalyst used) was added. The mixture was stirred at room temperature for 1 h and reprecipitated in methanol. The obtained powder was purified by Soxhlet extraction with methanol followed by hexane and finally with chloroform. The polymer was recovered as powder by reprecipitating in methanol and drying under vacuum.

Table S1: Molecular weight distribution of polymers

Polymer	M_n	M_w	PDI
**P1**	5594	9396	1.67
**P2**	4448	5892	1.32
**P3**	15576	20325	1.30

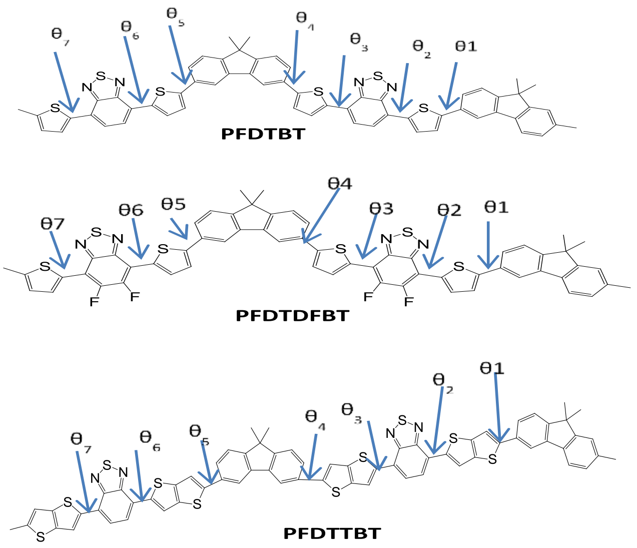

Figure S1: Dihedral angles of all the polymers

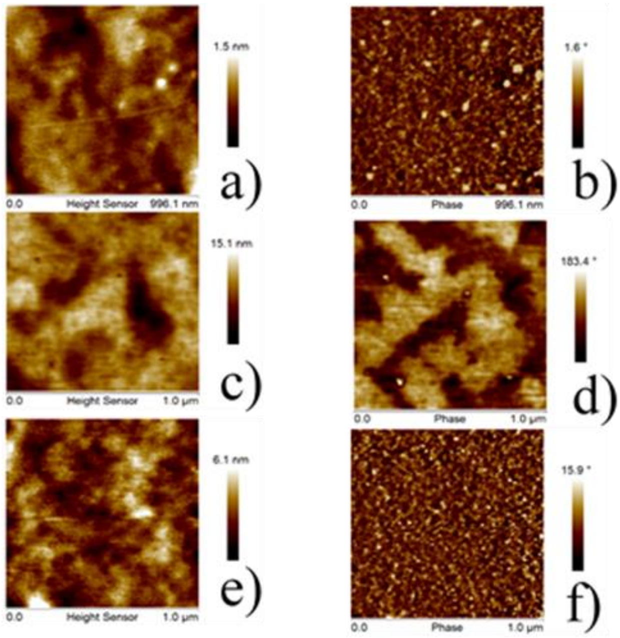

Figure S2: AFM topography images of blend of PC70BM with polymers **P1** (a), **P2** (c), and **P3** (e), respectively. Phase images of blends of PC70BM with **P1** (b), **P2** (d), and **P3** (f).

SCLC Mobility:

SCLC, hole mobility was measured using a hole only device configuration of ITO/PEDOT:PSS/DONOR:PCBM/Au by taking the dark current density in the range of 0–10 V and fitting the results to a space charge limited form using the Mott–Gurney equation, where current density is described by:

$$j = \frac{9}{8}\mu\varepsilon\varepsilon_0\frac{v^2}{d^3}$$

Where *J* is the current density, *d* is the film thickness of the active layer, μ is the hole or electron mobility, ε is the relative dielectric constant of the material (taken as 3), ε_o is the permittivity of free space (8.85×10^{-12} F m^{-1}), and *V* the applied voltage on the device. The mobility was extracted from the linear portion of the fitted curve.

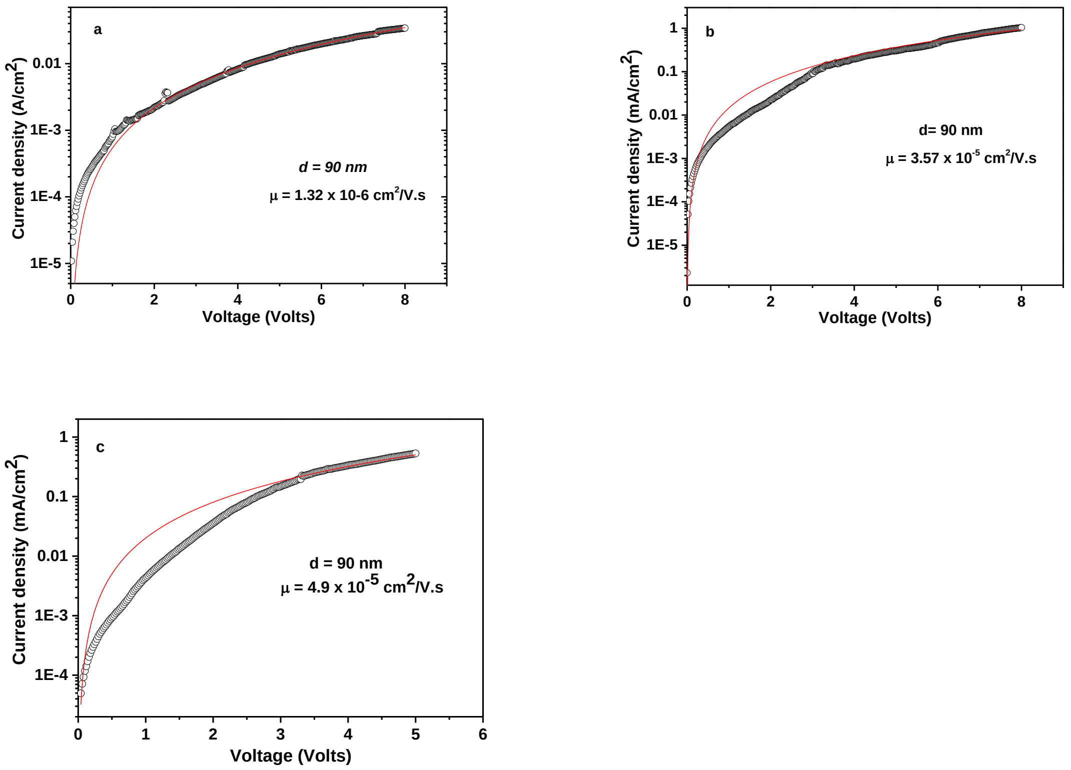

Figure S3: SCLC Hole mobility characteristics of (a) **P1**, (b) **P2** and (c) **P3**.

References

[1] K. Ranjith, S. K. Swathi, P. Kumar, and P. C. Ramamurthy, "Solar Energy Materials & Solar Cells Dithienylcyclopentadienone derivative-co-benzothiadiazole : An alternating copolymer for organic photovoltaics," *Sol. Energy Mater. Sol. Cells* 2012, 98, 448–454.

[2] S. Doi, "Molecular architecturing of a small two dimensional A-D-A molecule for photovoltaic application Vinila Nellissery Viswanathan, Arul Varman Kesavan, Praveen C Ramamurthy Department of Materials Engineering, Indian Institute of Science, Bangalore, 560012.," MRS Advances 2016, 1, 2917–2922.
